# Research on Continuously Tunable Carbon Nanotube Mode-Locked Fiber Laser

**DOI:** 10.3390/mi17040455

**Published:** 2026-04-07

**Authors:** Zhengyu Yang, Fei Wang, Pingping Xiao

**Affiliations:** 1 Department of Artificial Intelligence and Information Engineering, Yichun University, Yichun 336000, China; zyyang28-c@my.cityu.edu.hk (Z.Y.); ifei9999@163.com (F.W.); 2 Department of Systems Engineering, City University of Hong Kong, Hong Kong 999077, China

**Keywords:** tunable mode-locked laser, carbon nanotube saturable absorber, erbium-doped fiber, ultrafast, wavelength tuning, optical communication

## Abstract

This paper demonstrates a C-band continuously tunable mode-locked fiber laser based on a carbon nanotube saturable absorber (CNT-SA) and a commercial broadband tunable filter. The laser operates in the C-band with a continuous tuning range of 37.3 nm from 1532.6 nm to 1569.9 nm. The erbium-doped fiber (EDF) has a wide gain range, enabling the laser to achieve ultrafast mode-locking. Meanwhile, the tunable filter offers a broad wavelength selection range. This continuously tunable mode-locked fiber laser features a simple structure and a broad operating wavelength range, making it highly suitable for applications in optical communication, sensing, and laser processing.

## 1. Introduction

Mode-locked fiber lasers equipped with ultrashort pulse generators are indispensable tools for scientific research and commercial applications, including fiber optic communication systems, sensing, and materials processing [1]. Passive mode-locking is a widely applied technology for generating ultrashort pulses in fiber lasers. This technology utilizes actual or virtual nonlinear saturable absorbers (SAs) to introduce optical modulation into the laser cavity, thereby generating periodic pulse sequences from the continuous wave (CW) output [2]. However, techniques such as virtual SA-based nonlinear polarization rotation effects, nonlinear magnifying ring mirrors, or nonlinear halo mirrors are vulnerable to external perturbations due to their polarization dependence. Semiconductor saturable absorber mirrors (SESAMs) have been the most commonly used SAs, but their complex and costly manufacture and limited operational bandwidth have presented downsides. Their recovery times are often in the picosecond range, which can limit the generation of sub-picosecond pulses. Moreover, their performance is sensitive to temperature fluctuations and optical damage thresholds, which can compromise long-term reliability in high-power applications [3,4,5].

In recent years, carbon-based nanomaterials, notably single-walled carbon nanotubes (SWNTs) and graphene, have attracted significant attention as promising alternatives. These materials offer a unique combination of ultrafast recovery times (on the order of hundreds of femtoseconds), broad operational bandwidths, high optical damage thresholds, and compatibility with fiber integration. The saturable absorption in SWNTs arises from Pauli blocking, where photon absorption bleaches the valence band, leading to intensity-dependent transmission. The operational wavelength of SWNT-based SAs is primarily determined by their diameter distribution; for instance, tubes with diameters between 1.0 and 1.5 nm exhibit resonant absorption peaks in the 1550 nm region, aligning perfectly with the telecommunication C-band [6,7]. This tunability via diameter selection, coupled with the possibility of solution-based processing and thin-film fabrication, makes SWNTs highly versatile for photonic devices.

Simultaneously, the demand for wavelength-tunable ultrafast sources has grown considerably [8]. New mode-locked fiber lasers are highly desirable for various applications owing to their flexible output wavelengths, and broadband tunable mode-locked fiber lasers, in particular, hold significant practical value. Tunability enables wavelength-division multiplexing (WDM) in communications, multispectral sensing, and selective excitation in spectroscopy. Traditional tuning methods in fiber lasers often rely on intracavity birefringence filters or fiber Bragg gratings (FBGs). However, since the wavelength selection resulting from intracavity birefringence is random, it is challenging to precisely control the operational wavelengths through birefringence manipulation. Additionally, a common approach to generating ultrafast pulses is to employ fiber Bragg grating (FBG) filters for tuning the wavelength and pulse duration. However, the capacity of FBGs to adjust these parameters is restricted by their relatively narrow reflection bandwidth [9,10,11].

This work addresses these challenges by proposing and demonstrating a hybrid architecture that combines a broadband, cost-effective SWNT-based SA with a commercially available tunable optical filter. This configuration decouples the mode-locking mechanism from the wavelength selection process. The SWNT-SA ensures robust and self-starting mode-locking over a broad spectrum, while the tunable filter provides independent, continuous, and precise wavelength selection across the gain band of the erbium-doped fiber (EDF). This approach mitigates the trade-offs between bandwidth, tunability, and complexity, presenting a practical solution for applications requiring flexible and stable ultrafast pulses. In experiments, the laser achieved mode-locking between 1532.6 nm and 1569.9 nm using a carbon nanotube (CNT)-based SA. By incorporating the tunable filter to select the desired wavelength and leveraging the wide gain of the EDF at 1550 nm, the laser can achieve mode-locking over a wide range of wavelengths.

## 2. Experimental Setup

**Figure 1:**
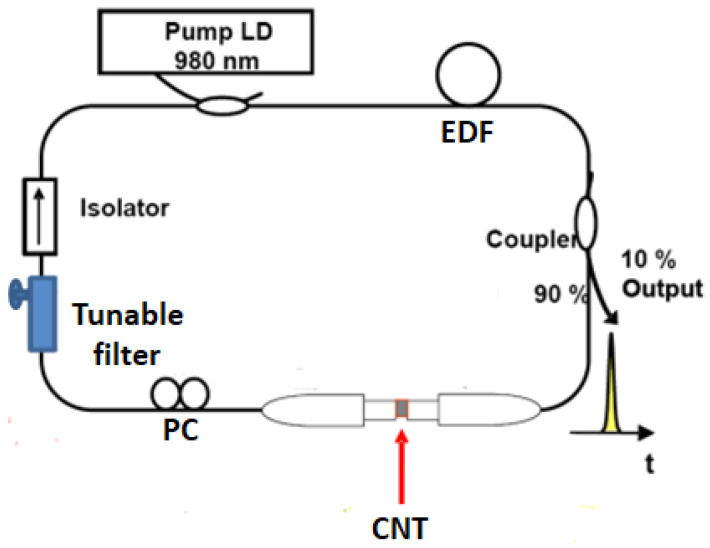
Schematic configuration of the proposed tunable mode-locked EDF ring laser.

The proposed tunable mode-locked EDF ring laser with a carbon nanotube-based saturable absorber (CNT-based SA) is depicted in Figure 1. The cavity of the ring fiber laser incorporates a gain fiber, specifically a 1.2 m EDF. A dispersion of −52 ps/(nm·km) at 1560 nm, along with a peak absorption ratio of 80 dB/m at 1530 nm, is measured in this EDF. The EDF undergoes forward-pumping by a 976 nm laser diode (LD). This pumping process is facilitated through a wavelength division multiplexer (WDM). The maximum pump power provided by the LD via the WDM is approximately 300 mW. To ensure unidirectional operation in fiber lasers, an isolator with a fiber pigtail is employed. Within the fiber laser cavity, a single-walled carbon nanotube/polyvinyl alcohol (SWNT/PVA) film is positioned between two fiber connectors to function as the mode-locking element [12,13,14,15]. Our manufacturing method ensures that the SWNT/PVA film thickness is suitable for controlled mode locking, reaching several tens of microns [15]. Therefore, inserting SWNT/PVA films between two fiber ferrules is a promising mode-locking method for fiber lasers. The softness of the SWNT/PVA polymer films makes the physical contact ferrule surfaces particularly advantageous. The film is held securely between the core centers without any air gap, allowing for quick and precise optical alignment. To counteract noise-induced pulse instability when the pump power surpasses the lasing threshold, the intracavity polarization is adjusted using a polarization controller (PC). The laser cavity is integrated with a tunable filter, which features a tunable wavelength range of 80 nm and a 3 dB bandwidth of 0.8 nm. The output signals are acquired through the 10% ports of a 10:90 optical coupler (OC).

A critical component for unidirectional lasing and isolation against back-reflections is a polarization-insensitive isolator, which is inserted directly after the EDF. The core mode-locking element is a saturable absorber fabricated from single-walled carbon nanotubes (SWNTs) embedded in a polyvinyl alcohol (PVA) polymer matrix. The fabrication protocol yields a freestanding, flexible SWNT/PVA composite film with a controlled thickness of several tens of micrometers. A small square (2 mm × 2 mm) of this film is carefully sandwiched between the ferrule ends of two standard FC/APC fiber connectors. The soft, compliant nature of the PVA matrix ensures excellent optical contact with the fiber cores, minimizing insertion loss and Fresnel reflections. This “sandwich” approach provides a robust, alignment-free, and integrable SA module.

To enable wavelength tuning, a commercially available tunable bandpass filter (with a 3 dB bandwidth of 0.8 nm and a continuous tuning range exceeding 80 nm) is incorporated into the cavity. Its central wavelength can be precisely adjusted via a manual micrometer or, prospectively, a motorized stage. A polarization controller (PC) consisting of three spools of fiber is placed in the cavity to adjust the state of polarization. This is essential for initiating and stabilizing mode-locking, as the SWNT-SA’s absorption has a slight polarization dependence, and the cavity’s nonlinear effects (e.g., nonlinear polarization rotation) can interact with the SA.

The majority of the cavity is constructed from standard single-mode fiber (SMF-28), with a total length of 9.4 m. The SMF-28 has a dispersion of 17.8 ps/(nm·km) at 1560 nm. The calculated total cavity length is approximately 10.6 m, corresponding to a fundamental repetition rate of ~19.3 MHz. The net cavity dispersion is estimated to be slightly anomalous at −0.13 ps^2^, which is conducive to the formation of conventional solitons. The laser output is extracted via a 10:90 optical coupler (OC), with the 10% port used for real-time diagnostics. The output pulses are characterized using an optical spectrum analyzer (Yokogawa AQ6370D), a 45 GHz digital sampling oscilloscope (Keysight DSAZ634A) coupled with a 12.5 GHz photodetector (Newport 818-BB-51F), and a commercial autocorrelator (Femtochrome FR-103WS) for pulse width measurement. The radio-frequency (RF) spectrum is analyzed using a 26.5 GHz signal spectrum analyzer (Keysight N9020B) to assess the stability and noise characteristics [16,17] (For detailed information about the equipment, please refer to the Appendix A).

## 3. Results and Discussion

The realization of high-performance passive mode-locked fiber lasers necessitates the use of high-quality CNT thin films [6]. Single-walled carbon nanotubes (SWCNTs) with diameters between 1 and 1.5 nm can be typically synthesized via the well-established method of catalytic chemical vapor deposition. This diameter range corresponds to a peak resonant wavelength of approximately 1550 nm [18]. The preparation process for SWNT/PVA nanocomposites involves two steps [19]. Firstly, several milligrams of SWNTs is dispersed in 50 mL of a 0.1% (*w*/*v*) aqueous sodium dodecyl sulfate (SDS) solution. This dispersion is achieved through strong ultrasonication at a frequency of 28 kHz and a power of 100 W for a processing time of 1 h. To minimize unwanted scattering losses from undispersed large SWNT bundles, the upper supernatant was collected. Subsequently, the carbon nanotube (CNT) dispersion and a 10 wt% aqueous solution of polyvinyl alcohol (PVA) were combined in a volume ratio of 1:2. The resulting mixture was then agitated for 3 h using a magnetic stirrer. The as-prepared carbon nanotube-polyvinyl alcohol (CNT-PVA) mixture is carefully poured onto a Petri dish. Subsequently, it is allowed to remain at ambient temperature for a period of one week to facilitate evaporation. As a result, a CNT-PVA thin film is formed. This thin film is then precisely sectioned into small square pieces with dimensions of 2 mm × 2 mm. These pieces were integrated as a saturable absorber by sandwiching them between two fiber-optic connectors. The CNT-based saturable absorber has a broadband absorption spectrum with a high absorption window in the C-band. For comparison, the negligible absorption spectrum of PVA over the entire wavelength range is also provided, relative to the absorption spectrum of SWNT.

**Figure 2:**
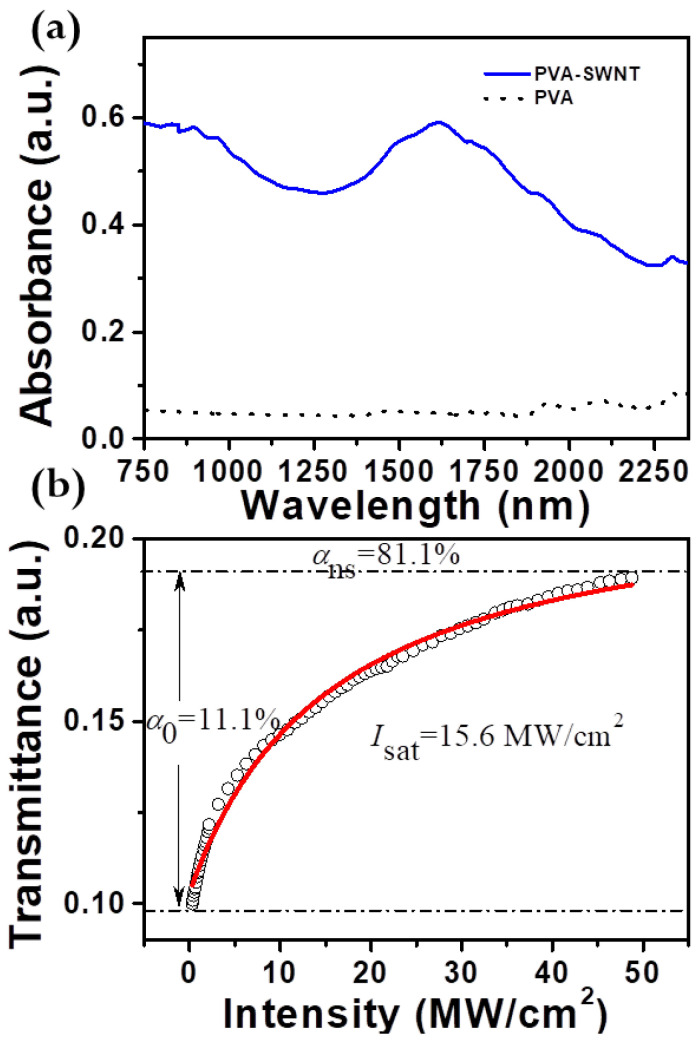
(**a**) The linear absorption spectrum; (**b**) The nonlinear saturable absorption of the prepared CNT-SA.

The nonlinear saturable absorption of the carbon nanotubes was characterized using a dual-arm measurement system, driven by a kind of passive mode-locked EDF laser. The laser output optical pulses feature a repetition rate of 40 MHz and a pulse width of 600 fs. To facilitate subsequent experimental operations, a 99:1 optical coupler was employed to divide the output pulse into two distinct branches. The saturated carbon nanotube absorber transmits 99% of the output power, and the remaining 1% is directly measured by a reference power meter. Figure 2a shows the nonlinear absorption measurements of the CNT saturable absorber at various incident pulse intensities. The red curve in Figure 2b represents the fitted curve for the saturable absorption equation.

(1) $$T(I)=1-∆T\cdot exp(-I/I_{sat})-A_{ns}$$

where T, ∆T, I, $I_{sat}$, $A_{ns}$ correspond to the transmittance, the modulation depth, the incident optical intensity, the saturation intensity, and non-saturable absorbance, respectively. The CNT saturable absorber, characterized from Figure 2b, exhibited a modulation depth of 11.1%, non-saturable absorption of 81.1%, and saturation intensity of 15.6 MW/cm^2^.

**Figure 3:**
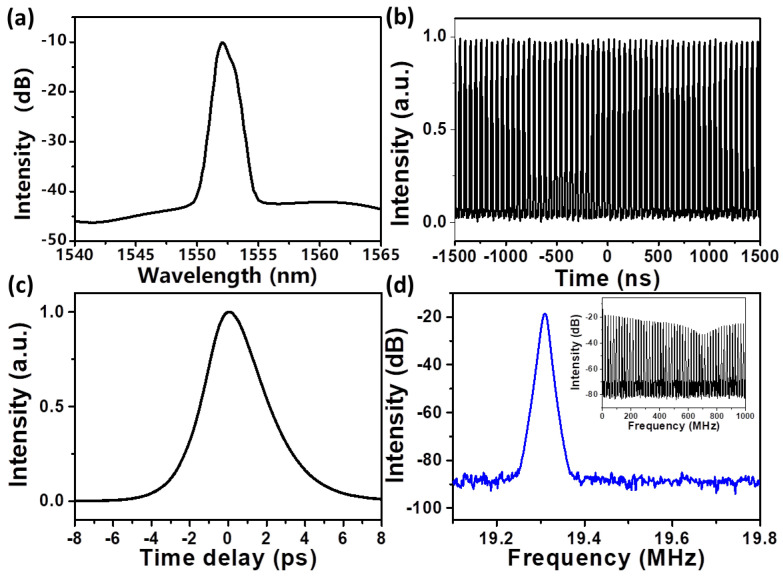
(**a**) The optical spectrum; (**b**) The oscilloscope trace; (**c**) The autocorrelation trace; (**d**) The RF spectrum of mode-locked output at 1552 nm. (The inset of (**d**) is the RF spectrum with a range of 1 GHz).

The experiment was conducted with an initial central wavelength of 1552 nm. When the pump power was increased to approximately 100 mW, stable mode-locked pulses were obtained solely through polarization controller (PC) adjustments. The output characteristics of the carbon nanotube (CNT) saturable absorber at this pumping power are summarized in Figure 3.

As shown in Figure 3a, the mode-locked optical spectrum exhibits a smooth envelope with no significant Kelley sidebands, despite the laser operating in a net anomalous dispersion regime. This spectral shape is attributed to the filtering effect of the intracavity tunable filter. The 3 dB spectral width was measured to be 1.1 nm. Figure 3b displays the corresponding pulse train, where the time interval between two adjacent pulses is 51.8 ns, corresponding to a fundamental repetition rate of 19.3 MHz, but this is only the basic repetition rate. The pulse profile was characterized using a commercially available autocorrelator. The resulting autocorrelation trace, presented in Figure 3c, indicates a pulse duration of approximately 2.6 ps. The slight asymmetry in the autocorrelation trace is likely due to a small residual chirp in the optical pulse, which can arise from imperfect dispersion compensation in the setup. The time–bandwidth product (TBP) was calculated to be 0.32, demonstrating that the conventional solitons generated by the anomalous-dispersion fiber laser are nearly transform-limited. The stability of the mode-locked laser is further confirmed by the RF spectrum depicted in Figure 3d. The spectrum shows a high signal-to-noise ratio (SNR) of 66 dB at the fundamental repetition rate of 19.3 MHz [18]. Furthermore, the measured repetition rate agrees well with the theoretical prediction based on the cavity length. Excellent stability of the single-wavelength mode-locked laser is confirmed by its wide-span RF spectrum (up to 1 GHz), which is characterized by low amplitude fluctuations [20,21].

**Figure 4:**
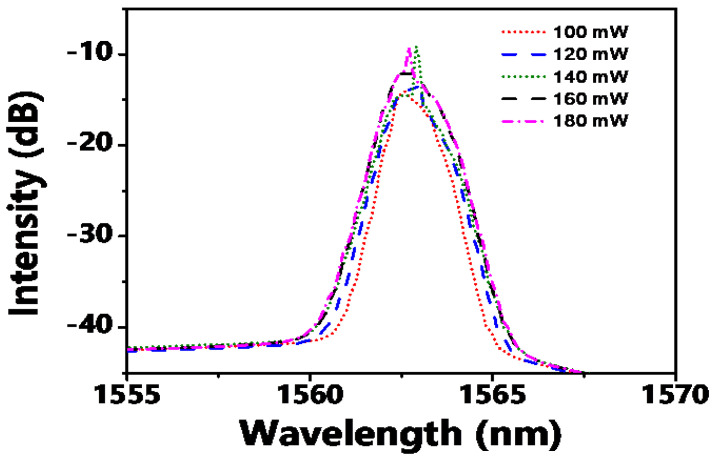
The spectral evolution with the increase in the pump power at 1562.6 nm.

The laser was set to operate at a power level of 100 mW and a wavelength of 1552 nm to attain mode-locking. To investigate the laser’s robustness and tuning capability, two parameter sweeps were performed: varying pump power at a fixed wavelength and varying the filter’s center wavelength at a fixed pump power. When the pump power was increased from the mode-locking threshold (~70 mW) to 180 mW at a fixed wavelength of 1562.6 nm, the optical spectrum evolved as shown in Figure 4. Initially, the spectrum broadened symmetrically with increasing power, a typical characteristic of soliton energy scaling. However, beyond ~150 mW, discrete, narrow spectral spikes emerged. These are identified as CW components or parasitic lasing lines. They arise because at high pump levels, the gain can saturate the SA non-uniformly across the spectrum, and the filter’s finite extinction ratio may allow a competing CW mode to lase. Crucially, it was found that by fine-tuning the PC, these spikes could be completely suppressed, restoring a clean mode-locked spectrum [20]. This demonstrates that the polarization state is a key degree of freedom for maintaining stability under high-power operation, likely by optimizing the nonlinear interaction between the SA and the intracavity birefringence.

Figure 4 shows that the spectral breadth expands owing to the filter operating at the central wavelength of 1562.6 nm and the mode-locking process occurring at 1562.6 nm as the pump power escalates. With the progressive increase in pump power, some anomalous spikes emerge in the optical spectrum. These spikes are most likely induced by the presence of a continuous-wave (CW) component within the laser system. The CW component can disrupt the stability of the mode-locking mechanism, leading to the observed spectral irregularities. Nevertheless, it has been demonstrated through experimental verification that by readjusting the polarization controller (PC), these spikes can be effectively eliminated. As a result, stable mode-locking can be re-established, ensuring the generation of high-quality optical pulses [22].

**Figure 5:**
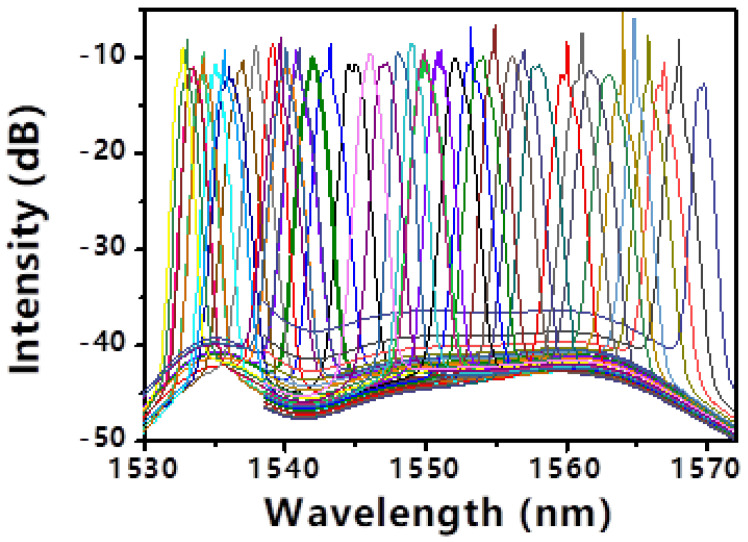
The Wavelength Tuning with a Fixed Polarization Controller.

**Figure 6:**
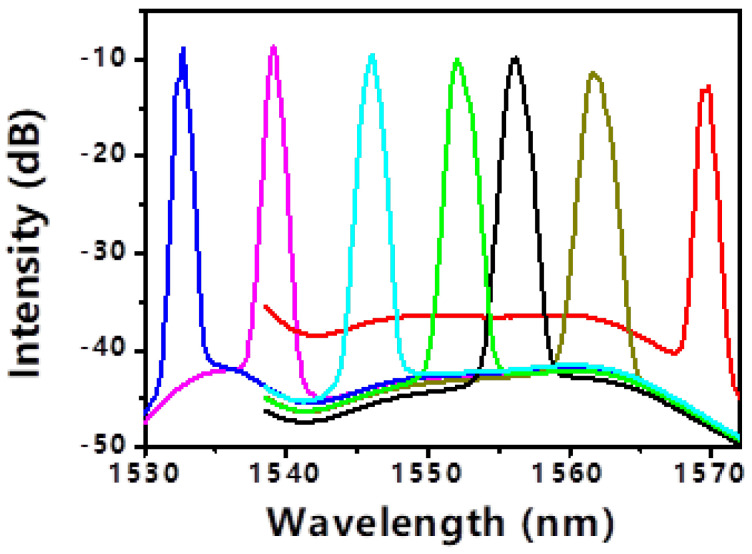
The mode−locked optical spectra without apparent spikes during the wavelength tuning.

The central wavelength of the tunable filter was systematically adjusted across the C-band while maintaining a pump power of 100 mW. With the PC fixed in an arbitrary state, mode-locking could be obtained at various discrete wavelengths from 1532.6 nm to 1569.9 nm, as shown in Figure 5. Figure 5 displays all the tuning results. However, the gain bandwidth of the EDF limits the wavelength tuning range to 37.3 nm. To achieve stable mode-locking without spikes in the spectrum, it is necessary to optimize the polarization state of the PC. Figure 6 shows the mode-locked optical spectra without apparent spikes at 1532.7 nm, 1539.1 nm, 1546.1 nm, 1552.1 nm, 1556.2 nm, 1561.7 nm and 1569.8 nm wavelengths. Their corresponding 3 dB bandwidths are 0.4 nm, 0.6 nm, 1 nm, 1.1 nm, 1.1 nm, 1.2 nm and 0.8 nm, respectively. To solve the problem of unstable mode locking in the spectrum during wavelength tuning, the increase in pump power and polarization adjustment were used.

With the PC fixed in an arbitrary state, mode-locking could be obtained at various discrete wavelengths from 1532.6 nm to 1569.9 nm, as shown in Figure 5. However, the spectra often exhibited the aforementioned spikes, and stable locking was not achievable at all intermediate wavelengths. This highlights a limitation: the interplay between the filter’s transmission peak, the EDF’s gain profile (which rolls off beyond 1560 nm), and the cavity’s polarization state creates a complex stability landscape. To achieve spike-free, stable mode-locking across the entire range, the PC was re-optimized at each target wavelength. The results, depicted in Figure 6, show clean optical spectra at seven representative wavelengths: 1532.7, 1539.1, 1546.1, 1552.1, 1556.2, 1561.7, and 1569.8 nm. Their respective 3 dB bandwidths are 0.4, 0.6, 1.0, 1.1, 1.1, 1.2, and 0.8 nm. The variation in bandwidth is attributed to the wavelength-dependent gain of the EDF and the dispersion profile. The total continuous tuning range achieved with stable, single-pulse operation is 37.3 nm (1532.6–1569.9 nm). This range is ultimately limited by the gain bandwidth of the EDF, not by the SWNT-SA or the filter. The pulse duration, as verified by autocorrelation at several wavelengths, remained between 2.5 and 3.0 ps, with the TBP staying close to 0.32 [23].

The use of a standard SMF-based cavity with manual PC adjustment, while demonstrating excellent performance, introduces sensitivity to environmental perturbations (temperature, vibration). This is a common challenge in non-polarization-maintaining fiber lasers. As noted in the manuscript, replacing standard fibers with polarization-maintaining fibers (PMFs) throughout the cavity would be a logical and highly effective upgrade. A PMF cavity would lock the polarization state, making the mode-locking initiation and stability independent of PC adjustments and far more robust against external disturbances. This would transform the system from a laboratory prototype into a field-deployable device.

Another avenue for improvement lies in the SA itself. While the SWNT/PVA film performs admirably, the non-saturable loss of 81.1% is relatively high. Advanced nanotube purification techniques (e.g., density gradient ultracentrifugation, gel chromatography) could yield dispersions with fewer metallic nanotubes and smaller bundles, reducing scattering losses. Alternatively, integrating the SA directly onto a fiber end-face (e.g., via optical deposition or taper–fiber evanescent field interaction) could significantly lower insertion loss and improve power handling [24].

The tuning mechanism, currently manual, could be automated using a piezoelectric or micro-electro-mechanical system (MEMS)-based tunable filter, enabling rapid, programmable wavelength sweeping—a feature highly desirable for sensing and spectroscopic applications. To intuitively show the advantages of this work, Table 1 compares the performance of our laser with other tunable mode-locked fiber lasers based on novel saturable absorbers reported in recent years [9,10,11].

## 4. Conclusions and Discussion

This paper has successfully demonstrated a simple, cost-effective, and continuously tunable (37.3 nm) mode-locked erbium-doped fiber laser. The laser leverages the complementary advantages of a broadband carbon nanotube saturable absorber and a commercial tunable filter. The SWNT/PVA SA provides robust, self-starting passive mode-locking with ultrafast recovery, while the tunable filter enables independent and precise wavelength selection over a 37.3 nm range in the C-band (1532.6–1569.9 nm). The system generates nearly transform-limited pulses of ~2.6 ps duration at a 19.3 MHz repetition rate with excellent spectral purity and temporal stability (RF SNR > 66 dB).

The architecture’s simplicity, relying on readily available components, makes it highly attractive for practical deployment. The results underscore the maturity of carbon nanotube-based SAs as reliable components for ultrafast photonics [25]. While environmental stability can be further enhanced by adopting a polarization-maintaining cavity design, the present work solidly establishes the feasibility and performance of this hybrid tuning approach.

Future work will focus on implementing the proposed PMF-based cavity, integrating an automated tuning system, and exploring the laser’s application in specific domains such as frequency comb generation, nonlinear microscopy, and as a seed source for optical parametric amplifiers [26,27,28]. The principles demonstrated here are also readily transferable to other gain bands (e.g., 1 µm, 2 µm) using appropriate SWNT diameters or other low-dimensional nanomaterials like graphene or topological insulators, promising a versatile platform for next-generation tunable ultrafast sources [29].

## Figures and Tables

**Table 1:** Performance Comparison with Prior Published Work.

Aspect	Our Work	Prior Published Work
Tuning Mechanism	Decoupled (CNT-SA + commercial tunable filter)	Coupled (SA + birefringence/FBG filter)
Tuning Range	37.3 nm (1532.6–1569.9 nm, continuous)	<30 nm (discrete for most)
Integration Method	Simple SWNT/PVA “sandwich” (low loss)	Complex film deposition/taper fiber evanescent field
Component Type	All commercial (detailed specs provided)	Custom-made components (low reproducibility)
Temporal Stability	RF SNR > 66 dB (no pulse missing)	RF SNR 50–60 dB (partial wavelength instability)

## Data Availability

The raw data supporting the conclusions of this article will be made available by the authors on request.

## Abbreviations

The following abbreviations are used in this manuscript:

EDF	erbium-doped fiber
SAs	saturable absorbers
CW	continuous wave
SESAMs	Semiconductor saturable absorber mirrors
SWNTs	single-walled carbon nanotubes
FBG	fiber Bragg grating
CNT	carbon nanotube
LD	laser diode
WDM	wavelength division multiplexer
SWNT/PVA	single-walled carbon nanotube/polyvinyl alcohol
PC	polarization controller
OC	optical coupler
SDS	sodium dodecyl sulfate
SNR	signal-to-noise ratio
PMFs	polarization-maintaining fibers
TBP	Time–bandwidth product

## Appendix A. Equipment List

**Table A1:** Equipment List.

Equipment Name	Brand	Model	Key Parameters	City	Country
Tunable filter	Thorlabs	VF-1550-0.8	3dB bandwidth 0.8 nm, tuning range 1500–1580 nm	Newton	United States
Optical spectrum analyzer	Yokogawa	AQ6370D	Wavelength range 600–1700 nm, resolution 0.02 nm	Musashino	Japan
Photodetector	Newport	818-BB-51F	Bandwidth 12.5 GHz	Irvine	United States
Digital sampling oscilloscope	Keysight	DSAZ634A	Bandwidth 45 GHz	Santa Rosa	United States
Autocorrelator	Femtochrome	FR-103WS	For pulse width measurement	Santa Clara	United States
RF signal spectrum analyzer	Keysight	N9020B	Frequency range up to 26.5 GHz	Santa Rosa	United States
